# Effects of transcranial direct current stimulation on temperature and pain perception

**DOI:** 10.1038/s41598-017-03173-2

**Published:** 2017-06-07

**Authors:** Laura Mordillo-Mateos, Michele Dileone, Vanesa Soto-León, Angela Brocalero-Camacho, Yolanda A Pérez-Borrego, Ana Onate-Figuerez, Juan Aguilar, Antonio Oliviero

**Affiliations:** 1 FENNSI Group, Hospital Nacional de Parapléjicos, SESCAM, Toledo, Spain; 2 Centro Integral de Neurociencias, HM Hospital Puerta del Sur, Móstoles, Madrid Spain; 3 Neurosciences Department, San Bortolo Hospital, Vicenza, Italy; 4 Experimental Neurophysiology Group, Hospital Nacional de Parapléjicos, SESCAM, Toledo, Spain

## Abstract

Transcranial direct current stimulation modifies cortical excitability and in consequence some cerebral functions. In the present study we aimed to elucidate whether tDCS could affect temperature and pain perceptions in healthy subjects testing different stimulation parameters. A total of 20 healthy subjects were studied by means of quantitative sensory testing. Two different experiments were performed. First, we studied the effects of 15 minutes 2 mA anodal transcranial direct current stimulation applied over left M1 and parietal cortex in two separated sessions. Then, we tested the effects of 5 minutes tDCS over M1 by means of a sham controlled design to optimize the possibility to study minimal effects of tDCS using different polarities (cathodal and anodal) and intensities (1 and 2 mA). 2 mA anodal tDCS, when applied for both 15 and 5 minutes over the motor cortex, increased cold perception threshold. Conversely, motor cortex cathodal tDCS modulated cold perception threshold only when 1 mA intensity was used. M1-tDCS can modify the temperature perception; these effects are polarity and intensity dependent. As stimulation intensity seems critical to determine the effects, we suggest that for clinical application strong anodal tDCS (>1 mA) or weak cathodal tDCS (<2 mA) should be used for pain control.

## Introduction

Treatment of chronic pain (CP) is often difficult and satisfactory pain control is not always achieved. However, invasive motor cortex stimulation (MCS) and non-invasive brain stimulation (NIBS) techniques have emerged as a potential therapy and have been reported to be highly successful in a proportion of treated patients^1^. However, although MCS is generally effective in pain relief, a conspicuous number of patients are not-responders. A high number of not-responders is unwanted and favours the expansion of the less expensive NIBS techniques over the neurosurgical techniques.

Non-invasive methods of brain stimulation, including repetitive transcranial magnetic stimulation (rTMS) and transcranial direct current stimulation (tDCS) are able to induce long lasting effects within the human motor cortex^2–4^ and have been used to treat pain. Anodal tDCS over primary motor cortex (M1) promotes an increase in cortical excitability, while cathodal tDCS induces a reduction of cortical excitability^5^, furthermore it was shown that the longer the duration of tDCS, the longer the induced after-effects^6, 7^. As far as it concerns cathodal tDCS, increasing intensity from 1 mA to 2 mA can switch motor cortex excitability inhibition into excitation^8^. Also, as intensity increases, the induced electric field goes deeper into the brain so that it is conceivable that the orientation and distance of the axonal or dendritic-somatic axis with respect to the electrical field could affect the resulting biological and clinical effects unexpectedly^9^. Finally, it is important to consider that little variations of electrode size area, shape, or placement (montage) can strongly influence the “diffusion” of the current and the geometry of the induced DC fields into the brain^10–13^.

Many prior studies have evaluated the effects of tDCS on different temperature and pain perception in healthy volunteers. A list of the most relevant works on this issue is given in Table 1 ^14–21^. It should be noted a great divergence across studies about the effects of tDCS on the studied parameters. For this reason further research is warranted.

**Table 1:** Most relevant papers about the effects of tDCS on temperature and pain perception in healthy volunteers.

Article	Polarity	Stimulation Intensity and duration	Evaluation protocol	Results	Target
Borckardt *et al*.^14^	Anodal Sham HD-tDCS	2 mA, 20 minutes	QST	Anodal HD-tDCS decreased heat and cold perception thresholds with no effects on heat pain threshold and just a small effect on cold pain threshold	M1
Bachmann *et al*.^15^	Anodal Cathodal Sham	1 mA, 15 minutes	QST	Cathodal tDCS increased cold perception threshold	M1
Grundmann *et al*.^16^	Anodal Cathodal Sham	1 mA, 15 minutes	QST	Cathodal tDCS increased cold detection thresholds in both hands and warm detection thresholds only in the contralateral hand	S1
Zandieh *et al*.^17^	Anodal Cathodal Sham	2 mA, 15 minutes	Cold Pressor Test	Anodal tDCS led to increment in pain threshold	M1
Jurgens *et al*.^18^	Anodal Cathodal Sham	1 mA, 15 minutes	QST	Neither anodal nor cathodal tDCS significantly were able to change somatosensory and pain perception	M1
Mylius *et al*.^20^	Anodal Cathodal Sham	2 mA, 20 minutes	QST	Anodal tDCS led to an increase of tolerance to heat pain	DLPC
Csifcsak *et al*.^21^	Anodal Cathodal Sham	1 mA, 10 minutes	Laser evoked potentials (LEP)	Cathodal tDCS significantly reduced the amplitude of N2 and P2	M1
Boggio *et al*.^22^	Anodal Sham	2 mA, 5 minutes	Peripheral electrical stimulation (PES)	Anodal tDCS of M1 increased both perception and pain thresholds, whilst stimulation of the DLPFC increased pain threshold only.	M1 DLPC V1

Quantitative sensory testing (QST) is a standardized method that allows the evaluation of temperature and pain perceptions by means of different kinds of thermal stimuli (warm, hot inducing pain, cold and cold inducing pain) thus providing information about each of the physiological pathways involved^22–24^. More in detail, QST provides the opportunity for assessment for Aδ-fibres and C-fibres that are involved respectively in temperature cold and warm perception and nociception (cold pain and hot pain)^25–27^.

To elucidate whether tDCS could affect temperature and pain perceptions in healthy subjects, we performed a double-blind study in which we evaluated the effects of 15 min 2 mA-tDCS on temperature and pain perception as measured by QST: we chose this tDCS protocol since it is commonly used to obtain pain relief in clinical applications^28^. In order to disclose a possible site-specificity of tDCS effects we applied the neuromodulatory protocol over M1 and parietal cortex (Pcor). Moreover, in a separate experimental session, we tested the effects of a very short tDCS protocol (5 min, cathodal and anodal, M1) by using a placebo-controlled double blind study to disclose the polarity and intensity dependency of the effects of tDCS on thermal and pain perceptions. Contemporary, by means of these experiments, we aimed to test which QST parameters can be used as a biomarker of the effects of tDCS, possibly to be used in future study about its application in pain treatment.

## Methods

### Subjects

Ten healthy volunteers (7 women, 3 men, mean age 31.9 ± 4.9 (SD) years, range 27–41 years) were studied by applying anodal tDCS 2 mA for 15 minutes over two different cerebral targets, M1 and Pcor. Moreover, eleven healthy volunteers (9 women, 2 men, mean age 31.5 ± 7.5 (SD) years, range 24–39 years) were studied with a very short duration tDCS protocol using a sham controlled design. Just one subject participated in both experimental protocols. All subjects gave informed consent prior to participation; the study was approved by the Ethical Committee for Clinical Research (Toledo, Spain) and was conducted in accordance with the Declaration of Helsinki.

### Experimental design

**Figure 1:**
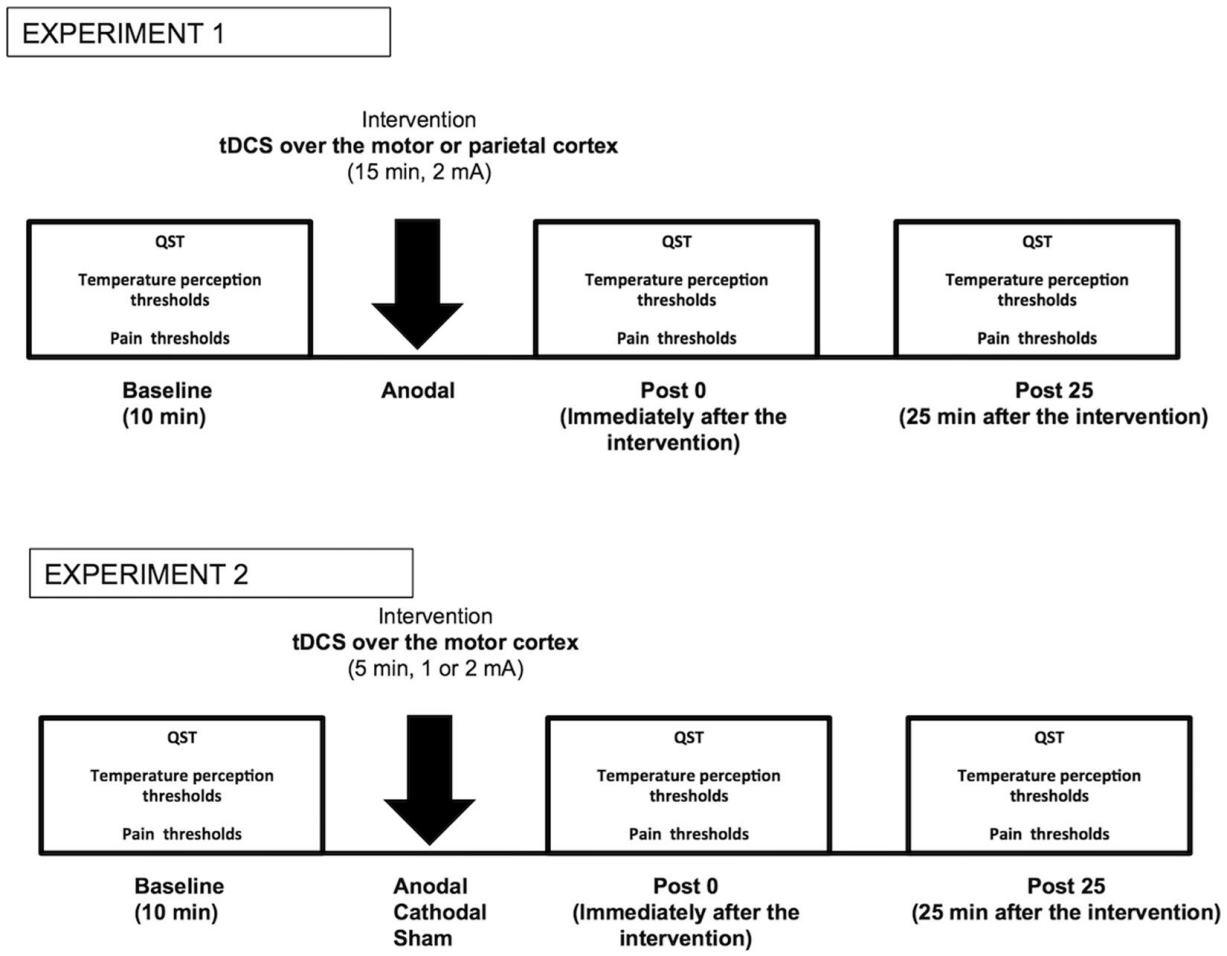
Schematic representation of the experimental setup.

Subjects were seated in a comfortable chair, with their arms and the whole body at rest. Sensory testing was evaluated prior to, immediately after, and 25 minutes after tDCS (times pre, post 0, and post 25, respectively). See Figure 1 for a schematic representation of the experimental protocols. The experimental protocols were randomly administered across the subjects and were separated by at least one week. The entire testing algorithm (temperature perception testing and pain thresholds) lasted for an average time of 12 minutes.

### Motor cortex localization: Transcranial Magnetic Stimulation

Transcranial magnetic stimulation (TMS) was performed with a Magstim 200 magnetic stimulator (Magstim Company, Whiteland, Dyfed, UK) and a figure-of-eight magnetic coil (diameter of one winding, 70 mm; peak magnetic field, 2.2 Tesla). The magnetic stimulus had a biphasic waveform with a pulse width of about 300 μs. During the first phase of the stimulus, the current in the center of the coil flowed towards the handle. The coil was placed tangentially on the scalp inducing a posterior-anterior current in the brain. We determined the optimal position for activation of the right first dorsal interosseous (FDI) by moving the coil in 0.5 cm steps around the presumed motor hand area of the left motor cortex. The site where stimuli of slightly suprathreshold intensity consistently produced the largest motor evoked potentials (MEPs) with the steepest negative slope in the target muscle was marked as the “hot spot”.

### Parietal cortex localization

To localize the optimal position of the electrodes to stimulate parietal cortex, we used as reference the motor hand area of the right first dorsal interosseus (FDI) localized by TMS (hot spot) and moving 2 cm posteriorly in the parasagittal direction^29^.

### Experiment 1: Comparing the effects of the anodal tDCS over the motor and parietal cortices

We tested the effects of a protocol similar to those commonly used to obtain pain relief in clinical applications^28^ (anodal tDCS, 15 min, 2 mA, M1 or Pcor) on two different cortical targets. With this experiment, we tested if there are effects on QST parameters that can be used as a biomarker of the effects of the tDCS. Furthermore, we tested the effects over the parietal cortex as some studies suggest that this target can be used for pain control^23^.

It should be noted that this experiment was developed to test if Pcor stimulation is effective in obtaining pain control and changes in temperature perception in healthy humans. We compared this site of stimulation to a commonly used protocol in pain treatment (Anodal tDCS 15 min over M1) and for this reason we did not use, in this first experiment, sham stimulation as a control condition.

All the subjects underwent two different experimental sessions: (1) anodal motor cortex tDCS, 2 mA, 15 minutes, (2) anodal parietal cortex tDCS 2 mA, 15 minutes.

Following the skin preparation to reduce impedance, saline-soaked sponge electrodes (5 × 7 cm) were positioned over the motor/parietal cortex and contralateral orbit, using the hot spot (for motor cortex stimulation) or 2 cm posteriorly in the parasagittal direction (for sensory cortex stimulation) as the center of the cortical electrode. Stimulation was applied for 15 min at a current of 2 mA (8 s phade in/phade out for a total stimulation time of 916 s). The current intensity and duration were within the established safety limits^30^. For anodal tDCS, the anode was positioned above the motor cortical representation of the right FDI while the cathode was placed above the contralateral orbit.

### Experiment 2: Placebo-controlled study on polarity and intensity effects of tDCS over the motor cortex

In Exp 1, we used the M1 stimulation (commonly used protocol in pain treatment) as a control for the Pcor stimulation. As we demonstrated that Pcor stimulation was inefficient in obtaining a significant change in any studied parameters (see results), in Exp 2 we tested the polarity and stimulation intensity dependence of the effects of M1 stimulation. Thus, in Exp 2 we added a sham tDCS as a control condition, cathodal tDCS to test also a different polarity and two different stimulation intensities to test (1 mA and 2 mA).

We tested the effects of a very short tDCS protocol (5 min) using a sham controlled design to optimize the possibility to study minimal effects of tDCS over the motor cortex of different polarities (cathodal and anodal) and intensities (1 and 2 mA). For this, we evaluated how tDCS applied to the motor cortex for 5 min was able to induce a change in sensory and pain perception, as measured by QST. This study was sham-controlled.

All the subjects underwent five different experimental sessions: (1) anodal tDCS of 1 mA; (2) anodal tDCS of 2 mA; (3) cathodal tDCS of 1 mA; (4) cathodal tDCS of 2 mA; (5) sham tDCS. Following the skin preparation to reduce impedance, saline-soaked sponge electrodes (5 × 7 cm) were positioned over the motor cortex and contralateral orbit^12^, using the hot spot identified with TMS as the center of the cortical electrode.

Stimulation was applied for 5 min at a current of 1 mA or 2 mA (8 s phade in/phade out for a total stimulation time of 316 s). The current intensity and duration were within the established safety limits^30^. Sham stimulation involved the same electrode placement and duration as the real conditions; however the constant current was delivered for only 30 s. Most subjects experienced a mild tingling sensation at the site of electrode contact that was independent of polarity and usually subsided after a period of a few seconds. For anodal tDCS and sham stimulation, the anode was positioned above the motor cortical representation of the right FDI while the cathode was placed above the contralateral orbit. For cathodal tDCS the opposite montage was used.

### Quantitative sensory testing

QST was conducted by using a Thermal Sensory Analyzer TSA 2001-II (MEDOC, Ramat Yishai, Israel)^31, 32^. This device uses computer controlled Peltier elements to heat or cool a contact plate in the thermode to the desired temperature. The thermode area was 3 cm × 3 cm (9 cm^2^). The entire thermode stimulating surface was placed in contact with the skin-testing site and secured by a Velcro band without stretch. Cold and warm thresholds were measured by stimulating right thenar eminence. The initial resting temperature of the thermode was 32 °C and the rate of the temperature change was 0.5 °C/s down or up for cold and warm trials, respectively. Stimulus magnitude was defined in each trial as the difference between the final temperature and the resting temperature of the thermode. Room temperature was controlled and kept between 23–24 °C.

### Warm and cold threshold determination

The method of levels (MLE) was used, because its measurements have been shown not to be influenced by reaction time^33^. A single stimulus of predetermined magnitude was presented and the subject indicated, after the cessation of the stimulus, whether it was felt or not. The initial stimulus step size was predetermined at a step of 3  °C (above or below the starting temperature of 32 °C). Subsequently, stimuli were decreased by intermediate steps of 1  °C, until the subject gave a negative response. The subsequent stimuli were increased or decreased by fine search steps of 0.3 °C. The direction changed according to the response, increasing for negative response (not felt), decreasing for positive response (felt). Null stimuli were randomly included. Four negative responses were required, after fine search had begun, to terminate the test. Threshold was determined by taking the mean of all results during the fine search step. Thresholds were expressed as the difference of the obtained value with respect to the starting temperature of 32 °C.

### Heat and cold pain threshold determination

Cold pain thresholds were measured over five trials by decreasing the temperature at a rate of 1 °C/s until the subject indicated that the temperature became painful. The threshold for heat pain was measured over five trials in which the temperature was increased until the stimulus was perceived as painful. Pain thresholds were determined by taking the average of the five successive trials. Thresholds were expressed as the difference of the obtained value respect to the starting temperature of 32 °C. The order of heat and cold detection and pain threshold measurements was randomised across participants.

### Statistical Analysis

Cold and warm thresholds and cold and heat pain thresholds in baseline condition were compared using unpaired t test. For this comparison only the baseline cold and warm thresholds obtained for anodal (2 measures) and cathodal (2 measures) and sham stimulation protocols have been averaged. The cold and warm perception threshold values were computed across subjects for both real and sham conditions and for the three time points (baseline, post 0 and post 25).

For the experiment 1, the effects of motor and sensory cortex 15 minutes tDCS on thermal thresholds (warm and cold) and pain thresholds (hot and cold) were evaluated by means of three-way mixed model ANOVA with TIME (baseline, post 0 and post 25) as within-subject factor and LOCATION (M1 and Pcor) and MODALITY (cold perception, warm perception, cold pain and hot pain) as between-subjects factors, followed – in case of a significant interaction– by four separate two-way repeated-measures follow-up ANOVAs (TIME and LOCATION) for each studied modality. Again, when a significant interaction was found, two separate one-way repeated measures follow-up ANOVAs (TIME) were performed for each LOCATION. When significant main effects or interactions were found, Tukey’s honest significant difference test was used for post-hoc comparisons.

In the experiment 2, we evaluated the effects of 5 minutes anodal tDCS, cathodal tDCS and sham stimulation when two different intensities were used (1 and 2 mA). To achieve this, we performed two separate three-ways mixed model ANOVAs for 1 mA and 2 mA stimulations whit MODALITY (warm perception, cold perception, heat pain and cold pain) and STIMULATION (anodal, cathodal and sham) as between-subjects factors and TIME (baseline, post 0 and post 25) as a within-subjects factor, followed – in case of a significant interaction – by four separate two-way repeated-measures follow-up ANOVAs (TIME and STIMULATION) for each studied modality. Again, when a significant interaction was found, other three separate one-way repeated measures follow-up ANOVAs (TIME) were performed for each STIMULATION. When main effects or interactions were found, Tukey’s honest significant difference test was used for post-hoc comparisons.

## Results

**Figure 2:**
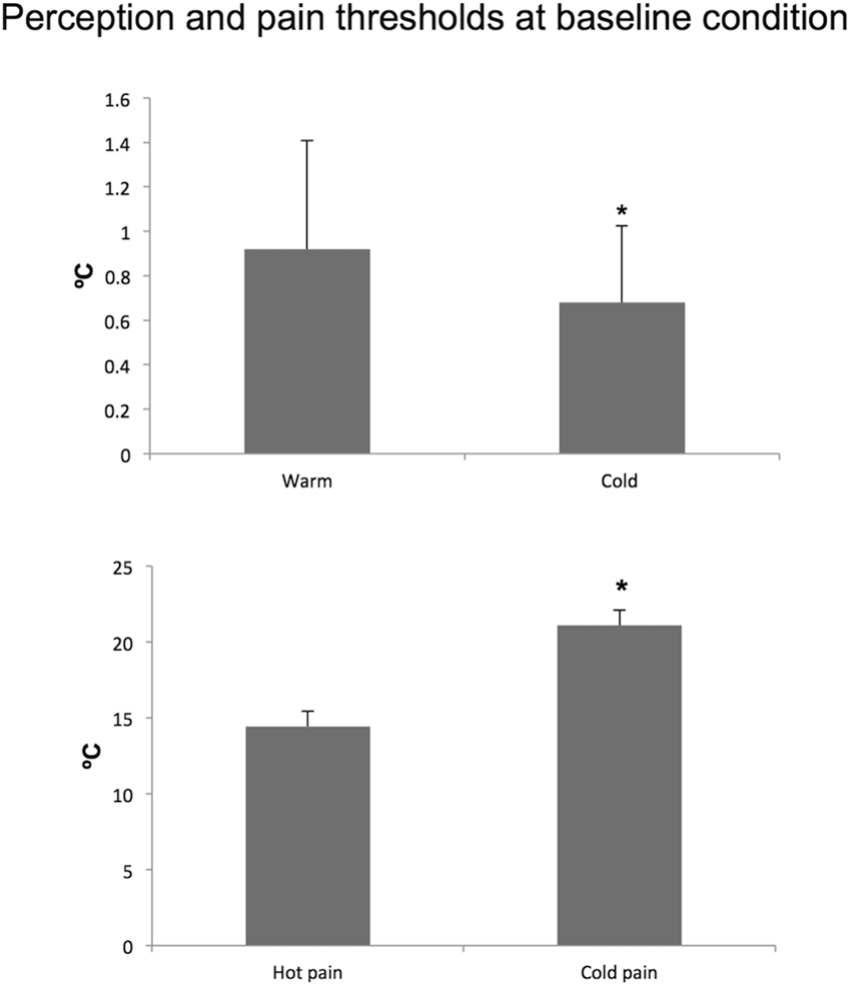
Temperature perception thresholds and pain threshold at baseline condition. Thresholds were expressed as the difference of the obtained value with respect to the starting temperature of 32 °C.

As previously reported by other authors using QST^17, 24^ baseline cold threshold was lower than warm threshold (0.68 ± 0.35 °C vs 0.92 ± 0.49 °C, p = 0.008 unpaired *t* test) (Fig. 2). Baseline cold pain threshold was higher than heat pain threshold (21.1 ± 5.2 °C vs 14.4 ± 2.1 °C, p < 0.001 unpaired *t* test) (Fig. 2).

### Experiment 1

At baseline, cold perception threshold, warm perception threshold, cold pain threshold and heat pain threshold did not differ between the 2 experimental sessions (paired t-tests, p > 0.5).

**Figure 3:**
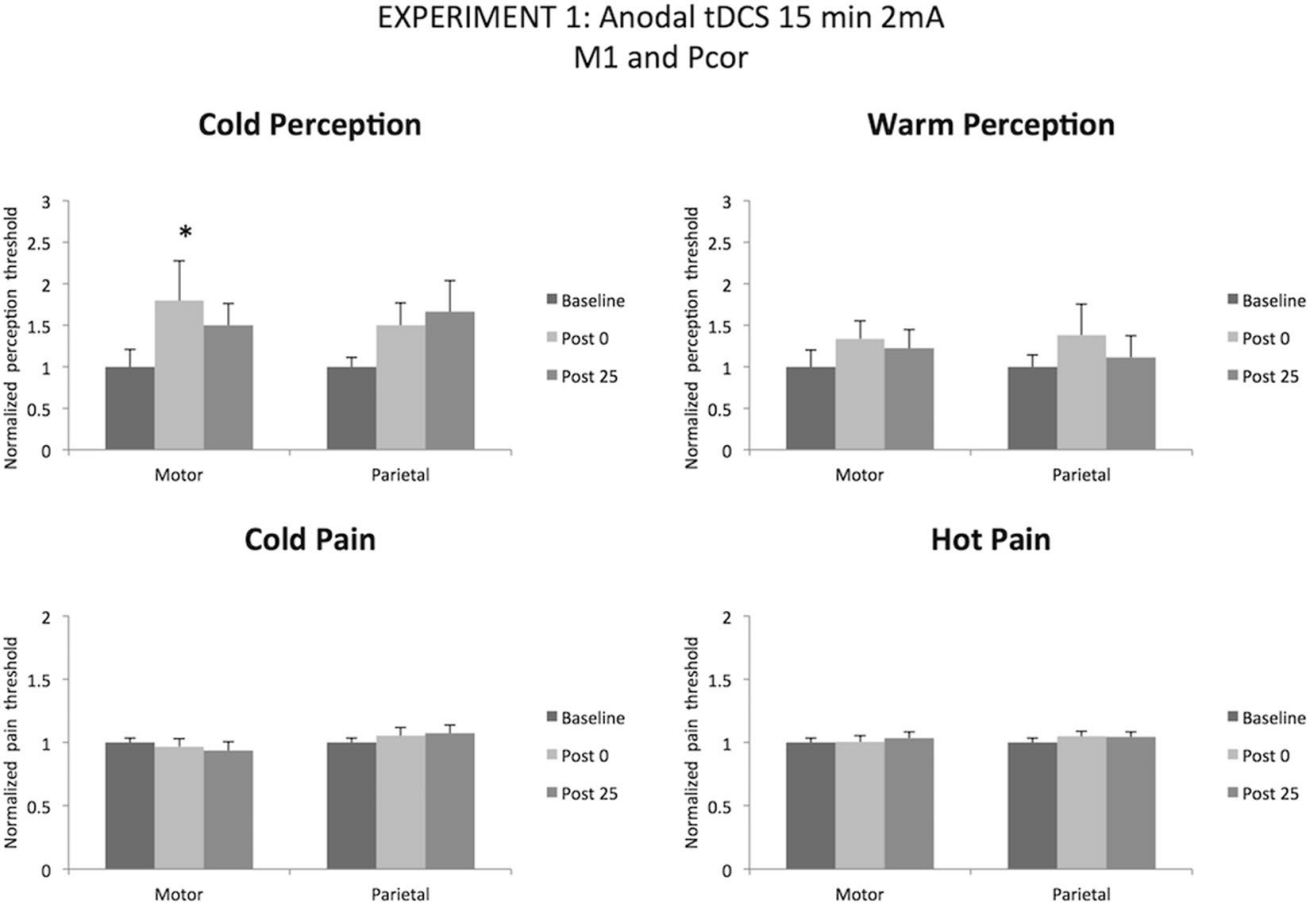
Temperature perception thresholds and pain thresholds for warm (hot) and cold perception (Anodal tDCS 15 min 2 mA over motor and sensory cortex). Error bars are standard deviations. *p < 0.05.

2 mA anodal tDCS, when applied for 15 min over the motor cortex, induced a change only in cold perception threshold but not in the other studied parameters (one-way follow-up ANOVA for cold perception: F_(2,18)_ = 3,68; p = 0.046)). In details, 15 minutes 2 mA anodal tDCS induced a significant increase in cold perception thresholds immediately after the end of the stimulation (p = 0.039) (see Fig. 3).

Moreover, 2 mA anodal tDCS when applied over the Pcor for 15 minutes induced no significant changes in all the studied parameters (one-way follow-up ANOVA for cold perception: F_(2,18)_ = 3,33; p = 0.058), we only observed a tendency to increase the cold perception (Baseline vs Post 25; p = 0.058).

### Experiment 2

At baseline, cold perception threshold, warm perception threshold, cold pain threshold and heat pain threshold did not differ between the 5 experimental sessions (paired t-tests, p > 0.5).

**Figure 4:**
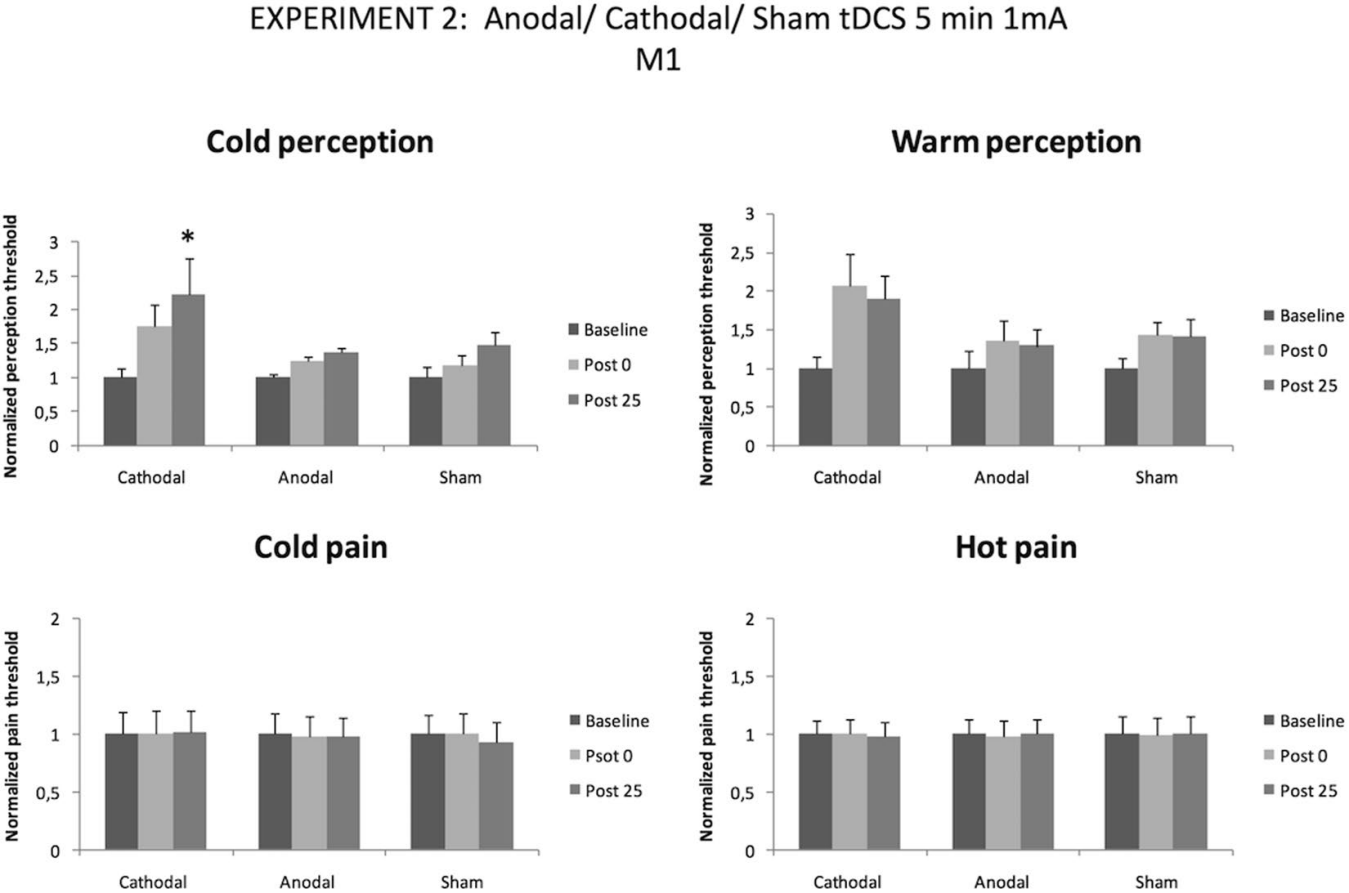
Temperature perception thresholds for warm and cold perception (anodal, cathodal and sham stimulation, tDCS 5 min 1 and 2 mA over motor cortex). Error bars are standard deviations. *p < 0.05.

Cold perception threshold was significantly modulated by tDCS (one-way follow-up ANOVA for cold perception: F _(2,20) = _4,60; p = 0.022). Particularly, when 1 mA was used, cathodal tDCS was able to increase cold perception threshold at 25 minutes after the end of the stimulation (p = 0.018) (see Fig. 4).

Anodal and sham 1 mA tDCS were able to change none of the studied parameters, and cathodal 1 mA tDCS was not able to change warm perception threshold, cold pain threshold and heat pain threshold.

**Figure 5:**
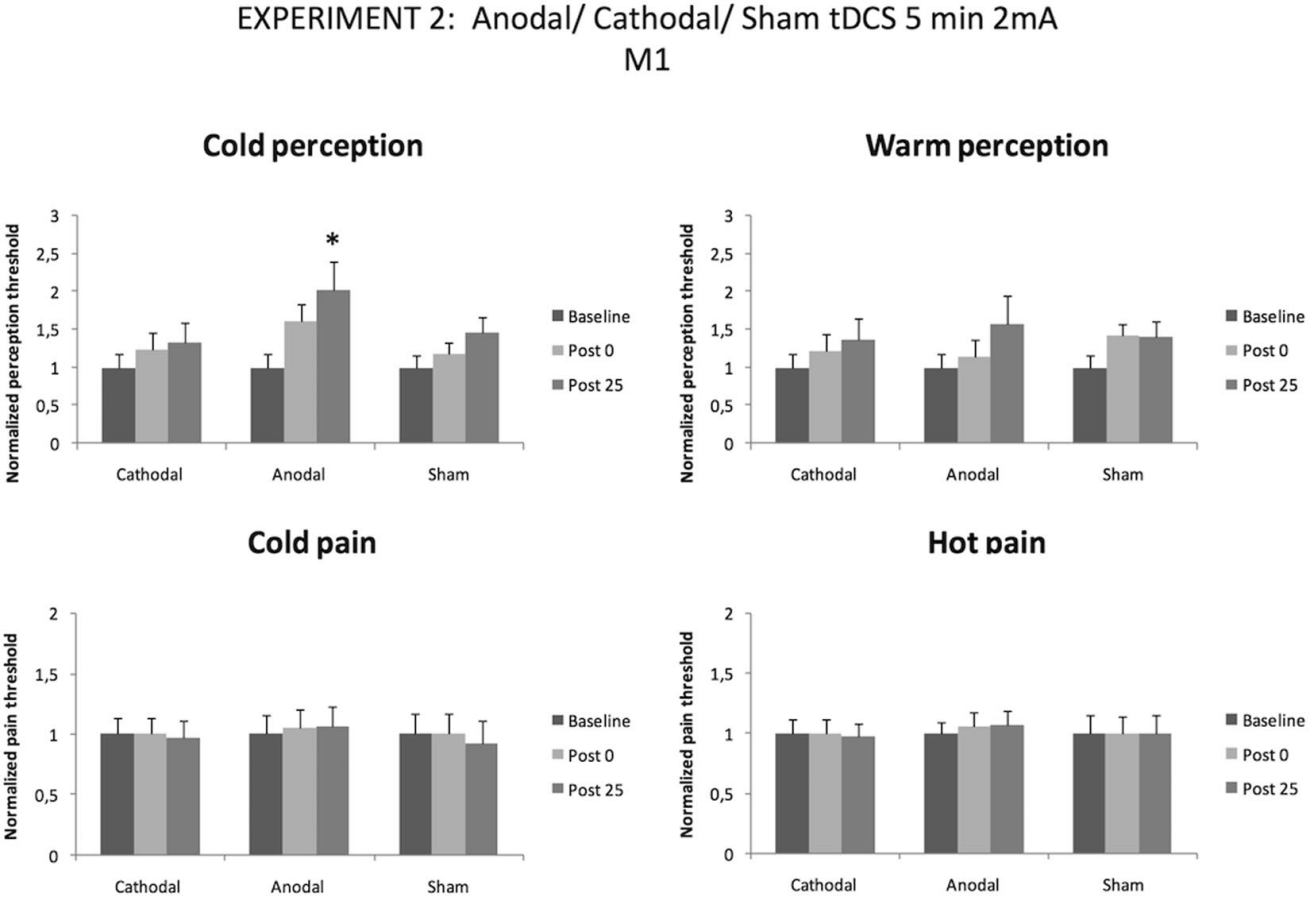
Temperature pain thresholds for hot and cold perception (anodal, cathodal and sham stimulation, tDCS 5 min 1 and 2 mA over motor cortex). Error bars are standard deviations. *p < 0.05.

When the intensity of tDCS was set at 2 mA, the only modality of stimulation able to increase cold perception threshold was anodal tDCS (one-way follow-up ANOVA for cold perception: F _(2,20)_ = 7,46; p = 0.004)). Particularly, cold perception threshold was significantly increased at 25 minutes after the end of the stimulation (p = 0.003) (Fig. 5 left upper panel).

## Discussion

The present study shows that motor cortex 2 mA-anodal tDCS, applied for both 5 min and 15 min, increased cold perception thresholds. Moreover the absence of effects when stimulating a non-motor cortex such as Pcor were demonstrated by applying 2 mA-anodal tDCS for 15 min. Hitherto, a single session of 15 min of anodal tDCS at 2 mA delivered over the motor or parietal cortex was unable to modulate pain perceptions. Moreover, when anodal tDCS was delivered with a smaller intensity (1 mA) and for 5 minutes, no effects on temperature and pain perceptions were found.

As far cold perception concerns, our findings are in line with previous studies. Indeed, Borckardt and co-workers, using a high definition tDCS montage, found that 20 min tDCS delivered at an intensity of 2 mA modified heat and cold sensory perception when anodal stimulation was used while no effects were found in heat pain threshold and just a small effect on cold pain threshold^14^. More in details, they reported that after stimulation lower temperatures were required to detect a change induced by cold stimuli. Furthermore, higher temperatures were required to detect a change induced by warm stimuli. On the other hand, we did not find any effects on the warm perception and cold and hot pain perception.

Moreover another study described that 2 mA anodal stimulation of the primary motor area can be utilized to alleviate cold pain perception^17^. However it should be considered that these groups found some effects on pain and this discrepancy respect our data could be due to the use of HD-tDCS^14^ that is characterized by a different spatial profile of induced brain current flow. On the other hand, Zandieh *et al*. (2012) evaluated the effects of 2 mA tDCS over cold pain threshold by using cold pressor test, so that the different evaluation methodology could have accounted for the slight different results^17^. In addition, it should be considered that the effects induced by tDCS are considerably affected by the presence of a large inter-subject variability as it was shown by similar studies using other forms of non-invasive brain stimulation^34^. In this light, the differences between our results and those from other groups could be also influenced by this large intersubjects variability and by the relatively little size of the studied cohorts.

Our data also suggest that anodal tDCS increases cold perception threshold by a dose-dependent mechanism in which intensity bigger than 1 mA is required. Furthermore we also observed that 5 minutes 1mA-cathodal tDCS applied over M1 significantly increased cold perception threshold, while no effects were found when using sham stimulation. This result was similar to the effects of 2mA-anodal tDCS. It should be noted that we found positive results only when tDCS was applied over M1 and not over Pcor. Since our main results consist in the modulation of not painful perception (i.e. cold perception), it could be expected that Pcor location was the most effective site of application. However Craig *et al*. found that the application of innocuous cold stimuli activates insula and not primary somatosensory cortex. Nevertheless, painful heat and cold stimuli activated the contralateral anterior cingulate cortex, contralateral primary motor, primary sensory cortex (S1), bilateral secondary sensory cortex, midinsular cortex, thalamus, and cerebellum^35^. Furthermore, functional neuroimaging also disclosed remote and widespread effects of tDCS and rTMS when focally applied to the primary motor or premotor cortex through fibres that project to remote cortical or subcortical structures involved in cognitive-emotional or discriminative aspects of sensorial experience and pain^3, 36^: thus it is not surprising that the stimulation of M1 is crucial in the modulation of thermal perception thresholds.

It is important to keep in mind that the tDCS we used cannot be considered “strictly” focal. Indeed, due to the large size of the tDCS electrodes (35 cm^2^) and the montage we used, it cannot be ruled out that when tDCS electrodes are placed over M1, the tDCS stimulation can affect also the parietal cortex (and vice versa). Indeed, a non focal effect of tDCS over M1 may spread to Pcor. This seems not to be the case, at least using the polarity (anodal) and the parameters we used here, as tDCS delivered over Pcor is less effective in modulating temperature sensory perception than M1 stimulation. Hitherto, we found a tendency to increase in the cold perception threshold after 2 mA anodal tDCS over Pcor (similar to the effects of M1 stimulation) that can be explained (may be not exclusively) by a spreading of the currents towards the M1.

Another interesting point from our results is that also 5 min cathodal stimulation induced an increase in cold perception threshold. Other groups found similar results, for example Bachmann *et al*.^15^ reported that cathodal stimulation of the primary motor area reduced sensitivity of A-fibers to somatosensory input (cold detection thresholds)^15^. Also, effects of the stimulation of the left S1 on thermal perception were found, with cathodal tDCS increasing cold detection thresholds in both hands and warm detection thresholds only in the contralateral hand^16^. In both cases tDCS was delivered at 1 mA intensity and cathodal stimulation was the most efficient when compared with anodal or sham stimulation.

Furthermore it should be considered that the net effect of motor cortex stimulation may be a mixed effect, as cathodal tDCS may also exert a “facilitatory effect” by deactivating inhibitory interneurons^37^ thus, hypothetically explaining the similar effects induced by cathodal and anodal tDCS in the present study.

We found no effect over pain thresholds. Ihle *et al*.,^38^, developed a study using functional imaging to explore the underpinnings of the previously suggested antinociceptive effects of tDCS over the motor cortex^38^. They found that neither cathodal nor anodal tDCS over the left M1 (1 mA, 15 minutes) significantly changed cortical nociceptive processing as a response to a heat pain paradigm when compared with sham stimulation. Only contrasting the interaction between responses to anodal and cathodal stimulation, It was found a substantial polarity-specific differences of regional brain activation after painful stimulation: anodal stimulation induced a decrease of regional Cerebral Blood Flow (rCBF), whereas cathodal stimulation resulted in an increase of rCBF in the hypothalamus, inferior parietal cortex, inferior parietal lobule, anterior insula, and precentral gyrus contralateral to the stimulation site^38^.

In sum, here we suggest the importance of evaluating the most efficient combination of intensity/polarity and site of stimulation for better results, since at the best of our knowledge this is the only work evaluating different intensities, polarities and cortical targets in the same study. As far as the parietal cortex stimulation, we found only mild effects. We cannot exclude that longer stimulation sessions or repeated sessions may have an effects on temperature perception. More studies would be done to better clarify these considerations.

We are aware that the findings obtained from healthy subjects have to be cautiously transferred to the patients. On the other hand, the effects of neuromodulation techniques in physiological conditions need to be understood before to think the multiple mechanisms that can condition the clinical response of the patients suffering for pain and sensory disturbances.

We can conclude that tDCS delivered over the motor cortex can modify the temperature perception and that these effects are polarity and intensity dependent. As stimulation intensity seems critical to determine the effects, we suggest that for clinical application strong anodal tDCS (>1 mA) or weak cathodal tDCS (<2 mA) should be used for pain control (in the attempt of reducing inconsistencies).
